# Might Depression, Psychosocial Adversity, and Limited Social Assets Explain Vulnerability to and Resistance against Violent Radicalisation?

**DOI:** 10.1371/journal.pone.0105918

**Published:** 2014-09-24

**Authors:** Kamaldeep Bhui, Brian Everitt, Edgar Jones

**Affiliations:** 1 Wolfson Institute of Preventive Medicine, Barts and The London School of Medicine and Dentistry, Queen Mary University of London, London, United Kingdom; 2 Institute of Psychiatry King's College London, London, United Kingdom; 3 King's Centre for Military Health Research, Institute of Psychiatry, King's College London, London, United Kingdom; Queensland University of Technology, Australia

## Abstract

**Background:**

This study tests whether depression, psychosocial adversity, and limited social assets offer protection or suggest vulnerability to the process of radicalisation.

**Methods:**

A population sample of 608 men and women of Pakistani or Bangladeshi origin, of Muslim heritage, and aged 18–45 were recruited by quota sampling. Radicalisation was measured by 16 questions asking about *sympathies for violent protest and terrorism*. Cluster analysis of the 16 items generated three groups: most sympathetic (or most vulnerable), most condemning (most resistant), and a large intermediary group that acted as a reference group. Associations were calculated with depression (PHQ9), anxiety (GAD7), poor health, and psychosocial adversity (adverse life events, perceived discrimination, unemployment). We also investigated protective factors such as the number social contacts, social capital (trust, satisfaction, feeling safe), political engagement and religiosity.

**Results:**

Those showing the most sympathy for violent protest and terrorism were more likely to report depression (PHQ9 score of 5 or more; RR = 5.43, 1.35 to 21.84) and to report religion to be important (less often said religion was *fairly* rather than *very important*; RR = 0.08, 0.01 to 0.48). Resistance to radicalisation measured by condemnation of violent protest and terrorism was associated with larger number of social contacts (per contact: RR = 1.52, 1.26 to 1.83), less social capital (RR = 0.63, 0.50 to 0.80), unavailability for work due to housekeeping or disability (RR = 8.81, 1.06 to 37.46), and not being born in the UK (RR = 0.22, 0.08 to 0.65).

**Conclusions:**

Vulnerability to radicalisation is characterised by depression but resistance to radicalisation shows a different profile of health and psychosocial variables. The paradoxical role of social capital warrants further investigation.

## Background

A process termed ‘radicalisation’ is proposed by governments across the world to explain how ordinary people with no history of criminality commit aggressive and terrorist acts against innocent civilians in their own country [1]. Conceptualised in social and psychological terms, this theory of radicalisation proposes a pathway which begins with the development of sympathies towards violent protest and terrorism through to active participation in violent terrorist acts [2], [3]. It is acknowledged that not all those with sympathies progress to become violent terrorists. Counter-terrorism efforts have been largely restricted to identifying high-risk individuals rather than understanding how to prevent radicalisation by intervening at an earlier stage of the process. Public health policies, research and interventions offer a different approach to health protection and intervene at the level of populations and *identify and reduce common risk* factors whilst *promoting protective factors and resistance* to rare diseases and damaging behaviour. Might this approach be applied to prevent radicalisation?

The recent waves of terrorism are sometimes understood as extreme religious movements with a particular focus on Islamic fundamentalism [4], but research into the propaganda spread by these groups suggests that it is political rhetoric disguised as religious ideology [5]. [6] Thus terrorism may be conceptualised as a powerful form of persuasion that induces fear and makes political gains by promoting dangerous and infectious ideas [6]. Taking this analogy further, Stares and Yacoubian propose that public health methods used to prevent the transmission of communicable diseases can also be deployed to inhibit the spread of terrorist philosophy [7], [8]. In their model, terrorist organisations are akin to a host, militant ideologies are the infectious agents, and settings such as prisons or even the internet act as vectors or vehicles for transmission. In fact, population approaches in public health are already being used to combat bio-terrorism and war [9], [10], homicide, violence, self-harm and suicide [11]. To advance this approach to prevent radicalisation we require a better understanding of risk and protective factors.

Among the conditions implicated in the radicalisation process are a sense of inequity and injustice, perceived discrimination, marginalisation, and a lack of integration in wider society [12] [13]. These narratives of adversity, alongside migration experiences, have also been proposed as causing depression and psychosis [14]–[19]. Social explanations and psychiatric opinions abound when known terrorists face conviction [20], yet there are few empirical population studies that investigate these possible explanations for radicalisation and terrorism [21], [22]. One exception is an exploratory study of 52 teenage boys in Gaza that found depressive symptoms were common in supporters of ‘religio-political aggression’, whilst anxiety was heightened amongst Palestinian supporters of radical action [23]. Another interesting study used convergence of psychometric measures from 356 suicide-bombers, tapes of self-immolations of 15 terrorists, and 918 ‘zealots’ in order to isolate risk factors [24]. The emergent profile implicated susceptibility to ‘dogma-induced psychotic depression’ amongst many other social and political characteristics. A risk-assessment instrument developed in Canada explored common characteristics of terrorists from diverse backgrounds [25], [26]. This identified perceived grievances about victimization and injustice as risk factors, whilst contact with other extremists, social isolation, previous exposure to violence, commitment to acts of terrorism because of anger or because of sacred ideology about rewards of participating in terrorism were proposed as contextual factors. Strikingly, protective factors in this study included a change in attitudes and beliefs about the role of terrorism in their lives, condemnation of it, and a commitment to move away from such acts.

Social capital has been implicated in radicalisation and terrorism [27]. This concept captures the assets, resources or ‘capital’ available to individuals and groups; it has been defined as community cohesion and resilience resulting from a rich associational life based on a strong array of co-operative social networks [11], [28]–[31]. Poor health and depressive symptoms can be a consequence of low social capital, isolation and inequalities, as well as experience of hardship, such as discrimination [32]–[34].

This study tests hypotheses about depression, psychosocial adversity and social assets as risk and resiliency factors in the early phases of radicalisation when preventive interventions might make the biggest impact [35]. In our previous paper we described a new way of assessing sympathies for violent protest and terrorism (SVPT) [36]. SVPT were rare (present in less than 3% of a population sample) so the study had limited power to detect effects. And we had not considered degrees of condemnation of violent protest and terrorism (CVPT) as a marker of resistance or as protective factors. Furthermore, whilst developing an outcome based on factor analysis, we removed items relating to international relations and government policy. In this paper, we use all the outcome data and methods to refine and improve our measure of radicalisation including all the items originally generated by focus groups and refined by testing.

In this paper we identify modifiable protective and risk factors that might be used to address vulnerabilities. We hypothesise that depressive symptoms, psychosocial adversity and limited social assets are positively correlated with SVPT and negatively correlated with CVPT. Furthermore, in our previous paper, we found that continuous measures of depressive and of anxiety symptoms did not correlate with SVPT, but we did not test non-linear associations across different levels of SVPT and CVPT, and nor did we test different validated thresholds used for screening for depression.

In this paper we address these limitations and ask the following research questions:

Are depression, psychosocial adversity and limited social assets risk (associated with SVPT) or protective (associated with CVPT) factors?

What demographic and psychosocial characteristics confer protection by showing associations with CVPT?

Are there risk and protective factors that show trends, akin to dose response relationships, for differing levels of condemnation to sympathies for violent protest and terrorism?

## Methods Section

### Sample

We surveyed 608 people of Pakistani or Bangladeshi family origins, aged 18–45, of Muslim heritage and living in East London and Bradford. The study sample methods have previously been reported (http://www.plosone.org/article/info%3Adoi%2F10.1371%2Fjournal.pone.0090718) [36]. Bradford was chosen because a significant proportion of the Muslim population live in traditional and relatively isolated areas characterised by poverty and deprivation. These factors are often thought to generate dissatisfaction and grievances, for example, when expressed through conflict between gangs and during riots [30], [34]. East London with a substantial Muslim population offers a contrast, having greater cultural diversity and wider opportunities for employment. Individuals were recruited to the study by quota samples, a standard method for opinion polls that entails setting quotas for participation on a range of demographic factors [37]. The expected number of Muslim households in each output area was estimated using the 2001 Census and mid-year 2010 estimates, and then classified into high, medium or low concentration areas. A disproportionately stratified sample of 70 sampling points was drawn; the sample over-represented areas with high and medium concentrates of Muslim households to improve the efficiency of recruitment. Within each sampling point, a representative population of Muslims aged 18–45 was interviewed: quotas were set on age, gender, working status, and whether Bangladeshi or Pakistani origin.

### Data Collection

The data were collected by IPSOS-MORI Social Research Institute. All questions were piloted and refined following two sets of four interviews to check wording, sensitivity, and questioning styles. Interviewers from IPSOS-MORI have significant experience of research into sensitive topics including religion and terrorism. IPSOS-MORI employs a workforce of trained interviewers resident in the communities where the survey took place so they could frame questions sensitively about social, lifestyle, health and safety issues for young Muslims. The pilot work did not indicate difficulties. Although the option of language and gender specific interviews was available, it did not prove necessary. Questions were asked in a computer-assisted format with prompts and cues so that sensitive questions could be answered anonymously.

### Survey Questions

#### A measure of risks for and resilience against violent radicalization

There is currently no well-established measure of radicalisation at a population level. Therefore, as reported in our previous paper [36], we developed survey questions by a series of interactive focus groups with young people of Muslim heritage and with members of a community panel of religious and non-religious organisations. This preceded the survey. The focus group generated a spectrum of 16 acts of protest ranging from non-violent (one item) through to more and more extreme acts, and culminating in acts of terrorism; for example, use of suicide bombs to fight injustice or commit terrorist acts (see Tables S1 and S2). In the survey, subjects were asked to rate their sympathies or condemnation of these acts. All except two of these items were scored on a 7-point Likert scale from -3 (completely condemn) to +3 (completely sympathise). Thus the higher the score, the greater were the sympathies for violent protest and terrorism. Two questions asked about sympathies for or condemnation of the British government's decision to send British troops to Afghanistan and to Iraq. These were reverse-scored as, according to our focus group discussions during item generation, condemnation rather than sympathies would be consistent with a more radicalised perspective [4].

#### Risk and protective factors

Social capital can be measured in many ways and is known to be associated with violence [30], [31], suicide [38] and mental health [39]. To measure social capital, we asked about satisfaction with living in the area (very satisfied, fairly satisfied, neither satisfied or dissatisfied, fairly dissatisfied, very dissatisfied), number of years living in a particular area (<1 year, 1 to <2 years, 2 to <5 years, 5 to <10 years, > or  = 10 years), how many people in local area can be trusted (many, some, a few or none), and feelings of safety when alone in the area after dark (very safe, fairly safe, fairly unsafe, very unsafe). Answers were scored and summed so that a *higher* score reflected *higher social capital* (0–18). As a measure of social isolation and support, we asked about the number of contacts by telephone, email, or visit in the preceding two weeks by friends or relatives.

Understanding of political processes or ready access to political institutions might serve as protective factors as they offer a democratic and non-violent method to address perceived injustices. We assessed political engagement by asking about participation in 12 types of activity in the preceding three years. These questions were from the Department of Communities and Citizenship Survey [40]. These questions addressed voting in local council elections, political discussions, signing a petition, donations to a charity or campaigning organisation, payment of membership fees to a charity or campaigning organisation, voluntary work, a boycott for political, ethical, environmental or religious reasons, political views expressed online, attendance at a political meeting, donations to or membership of a political party, and participation in a demonstration or march. The total number of activities formed a measure of political engagement (scores from 0–12) [40].

We assessed perceived discrimination by asking four questions from the EMPIRIC study [33]; these asked about physical assault, damage to property and insults in the preceding 12 months, and any instance of unfair treatment at work where these events were thought to be related to race, skin colour, religion or ethnic background insults. The measure of threatening life events included injury, bereavement, separations, loss of job, financial crisis, problems with the police or courts, theft and major stressful events in the preceding 12 months (score 0–12) [41].

Given the emphasis on religiosity as an explanation, questions on religiosity were included. These questions asked about the importance of religion in everyday life (very important versus fairly important/not important/don't know) and frequency of attending a mosque (weekly or more, monthly or less frequently, or never). We also asked about a number of demographic characteristics (age, employment), place of birth.

#### Health Outcomes

To measure general functioning related to poor health, four Likert questions were adapted from the SF12 [42]. These self-report measures assessed general health, reduced activities because of physical or emotional problems, being limited by physical or emotional problems, and not being able to undertake any vigorous activities. They were each recoded to binary variables and summed so that a higher total score indicated poorer health (scores range from 0–4). Anxiety was measured by the Generalised Anxiety Disorder Assessment (GAD-7) [43] and depression by the Patient Health Questionnaire (PHQ-9) as total scores and as non-linear scores using validated thresholds [44].

#### Statistical Analyses

We calculated the number of people showing sympathies for or condemnation of violent protest and terrorism (Table S1: for each of the Likert items we show weighted and unweighted estimates). The weighting was for combined design effect and response weights to ensure the estimates were reflective of population level findings. Weighted calculations were undertaken in STATA 11.0 using the ‘pw’ command to provide robust standard errors and the procedure does not inflate the sample bases [45].

To explore whether there was evidence of distinct groups of respondents who exhibited varying degrees of radicalisation, a particular method of *cluster analysis* was applied to the data for the 16 radicalisation items [46]. In this study a *classification likelihood method* was used [46], [47]. The Bayesian Information Criterion was used to determine the number of cluster in the data. The clustering was carried out on the principal component scores from a principal components analysis of the original 16 item scores. The clustering was carried out using different numbers of principal component scores and the most stable solution found was the one with the three groups described in this papers. The cluster analysis was implemented in the *mclust* package in R. All other analyses were conducted in STATA (11.0).

A cluster analysis of the 16-item measure of sympathies for violent protest and terrorism produced a three-group solution: a group that was most sympathetic (group 3, n = 92), least sympathetic (group 1, n = 93), and a large intermediary group (group 2, n = 423). The values of the mean score for each group suggest the cluster labels reflect an ordinal variable for radicalisation, so the study has potential to identify dose-response relationships. (Table S2: the mean scores for each cluster are shown overall and for each of the 16 items).

For each of the proposed risk and protective factors, we calculated the total sample size for each response category, and show the total weighted prevalence (%) and standard error (se) for categorical variables; for continuous variables we provided and weighted mean and standard error (se).

After defining the three groups derived from cluster analysis, univariable weighted distributions health measures and potential risk and resiliency factors were calculated and charted for each of the three *cluster derived groups*. The three-group cluster derived variable was used as a three level outcome in multinomial logistic regression models (univariable and multivariable); the largest group, group 2 served as the reference category.

For the weighted multivariable model, all the variables were included in order to provide the most conservative model that would take account of potential residual confounding. The findings are shown as Risk Ratios, and 95% confidence intervals and probability values (RR, 95% CI, p). Religious practice and importance were included to test whether the findings were sustained independent of religiosity. A gender interaction term with depressive symptoms was considered given depression is more common in women. Model fit with and without the interaction term was assessed using the likelihood ratio test.

Our ‘a priori’ framework for interpreting the results proposed that any variable more commonly found in group 3 compared to 2 was associated with sympathies for violent protest and terrorism, and was therefore a risk factor for violent radicalisation; in contrast variables less common in group 3 compared with 2 would offer resistance or be like resiliency factors. Any variables that were more common in group 1 compared with 2 would be implicated as a correlate of condemnation of violent protest and terrorism and therefore would be interpreted as a offering resistance or resiliency; any variable less common in group 1 compared to 2 would then be implicated as undermining resistance.

#### Ethical approval

Ethical approval was from Queen Mary, University of London Research Ethics Committee (REC); additional legal advice influenced the wording of questions. The REC reviewed and approved all the questions, and the information sheets and consent forms. The study was subject to an independent monitoring committee chaired by a professor of ethics in the law department at Queen Mary University of London. No adverse incidents or issues were reported during data collection. Data were collected by individual interviews, administered on a laptop computer by interviewers. Consent was recorded by checking an appropriate box before proceeding with the computer-based survey.

## Results

### Demographic, psychosocial, and health characteristics

Table 1 shows weighted response rates for each of the demographic, psychosocial and health characteristics. The weighted data representative of the population show a sample composed mostly of the 26–35 year age group, who are mostly employed and born in the UK, living in London; for the majority, religion was reported as *very important* in their daily lives, a point supported at least weekly mosque attendance.

**Table 1:** Demographic, psychosocial & health characteristics.

			Weighted
**DEMOGRAPHICS**			
**Age group**	18–25	%	25.98
**(N = 599)**	26–35	%	48.91
	36–45	%	25.11
**Gender**	Men	%	54.45
**(n = 608)**	Women	%	45.55
**Single**	Yes	%	54.45
**(N = 608)**	No	%	45.55
**Employment status**	Employed	%	50.26
**(N = 608)**	Full time education	%	11.05
	Unemployed	%	9.92
	Permanent disability/retired	%	0.64
	Look after house		28.14
**City**	London	%	80.62
**(N = 608)**	Bradford	%	19.38
**ETHNICITY AND RELGIOSITY**			
**Ethnic Group**	Pakistani	%	46.65
**(n = 608)**	Bangladeshi	%	53.35
**Born in UK**	Yes	%	70.93
**(n = 608)**	No		29.07
**Importance of Religion to way of life**	Very	%	71.99
**(N = 608)**	Fairly, not or don't know	%	28.1
**Attending a place of worship: mosque**	Never	%	28.47
**(N = 604)**	Monthly or less	%	13.86
	Weekly or more	%	57.68
**PSYCHOSOCAL ADVERSITY AND SOCIAL CAPITAL**			
**Social capital score**		mean	7.61
**(N = 608)**		se	0.21
**Total social contacts**		mean	4.64
**(N = 608)**		se	0.21
**Political engagement**		mean	1.98
**(N = 608)**		se	0.12
**Discrimination**		mean	0.2
**(N = 608)**		se	0.04
**Life events**		mean	0.58
**(N = 608)**		se	0.07
**HEALTH MEASURES**			
**Generalised anxiety on GAD score**		mean	2.95
**(N = 562)**		se	0.47
**Depressive symptom on PHQ9 score**		mean	3.34
**(N = 527)**		se	0.5
**Poor Health Total Score**		mean	0.74
**(greater score = poorer health)**		se	0.11
**(N = 608)**			

**Figure 1:**
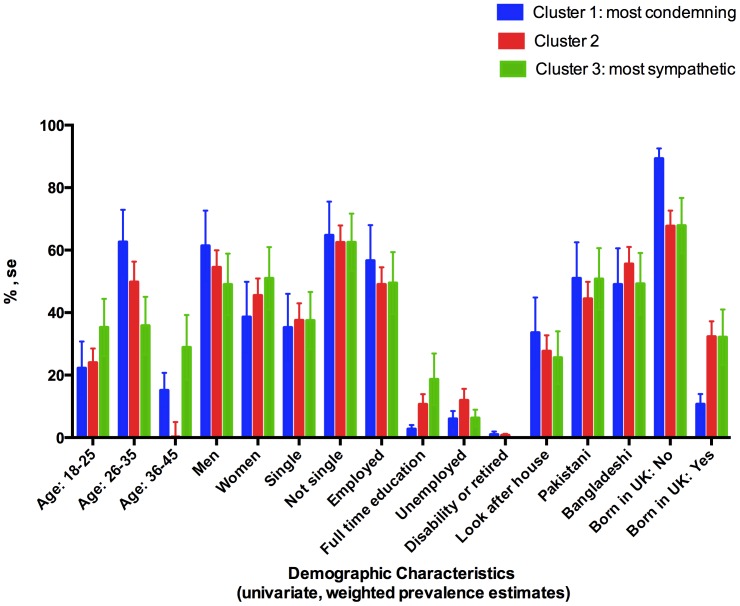
Demographic characteristics by clusters (univariable, weighted data).

**Figure 2:**
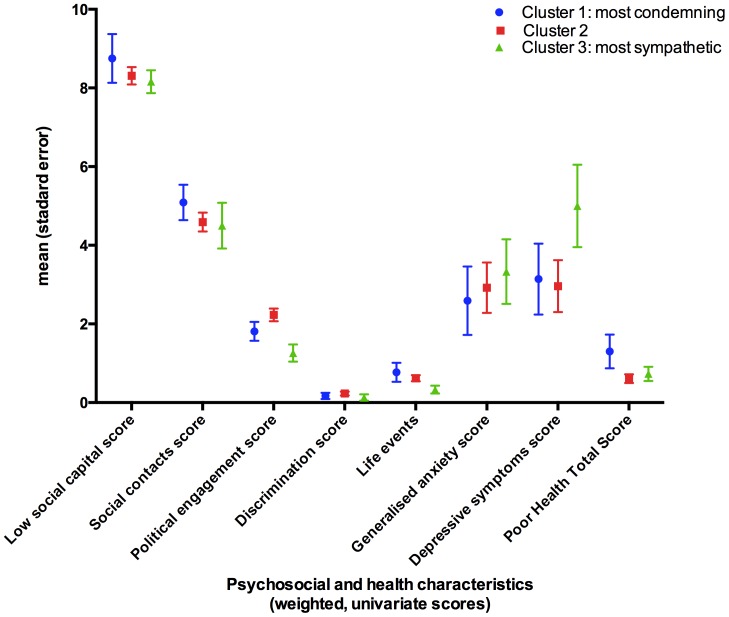
Psychosocial and health characteristics by clusters (multivariable, weighted data).

Weighted (unadjusted) population prevalence estimates of risk and protective factors were charted by the three groups derived by cluster analysis. The data for demographic characteristics are charted in Figure 1 as percentages and standard errors. Figure 2 shows the psychosocial and health characteristics.

In comparison with group 2, unemployment was less common in group 1 (RR = 0.23, 95%CI: 0.07–0.77, p = 0.02); social capital was poorer in group 1 (RR = 0.76, 95%CI: 0.63–0.92, p = 0.004; people in group 1 were less likely to be born in the UK (RR = 0.22, 95%CI: 0.08–0.65, p = 0.006). Among migrants, the number of years of residence in the UK did not show any relationship with cluster membership (unreported data).

In comparison with group 2, political engagement was poorer for group 3 (RR = 0.63, 95%CI: 0.44 to 0.9, p = 0.01) and depressive symptoms appear to be more prevalent for group 3 (weighted mean 5.00, 95%CI: 2.95 to 7.06) (weighted mean 3.14, 95%CI: 1.38 to 4.90). Psychosocial adversity and poor health appeared not to be relevant.

In order to address potential confounders, adjusted logistic regression models were built. This approach addresses potential confounding influences that might show associations in unadjusted analyses that are not sustained in multivariable analyses; or negative confounders may mask true associations in unadjusted analyses for these only to be revealed in multivariable analyses.

### Multivariable analyses (weighted)

Table 2 sets out the estimated risk ratios and associated confidence intervals for associations between cluster derived groups and demographic characteristics, psychosocial adversity, social capital and health measures, adjusted for ethnicity and religiosity. The comparisons are of group 3 with group 2, and of group 1 with group 2. Overall there was good model fit (R2 = 25.38, X2  = 85.51, df = 44, p = 0.0002),

**Table 2:** Multivariable Multinomial Logistic Regression comparing clusters three (most sympathetic) and cluster one (most condemning) with cluster two as the reference category (weighted).

		Cluster 1				Cluster 3			
		RR	LCI	UCI	P	RR	LCI	UCI	P
**DEMOGRAPHICS**									
Age groups	18–25	1				1			
	26–35	1.56	0.35	6.96	0.56	0.54	0.15	1.97	0.35
	36–45	1.4	0.33	5.87	0.65	0.72	0.14	3.64	0.69
Gender	Male	1				1			
	Female	0.26	0.06	1.15	0.08	2.92	0.78	10.98	0.11
Single	No	1				1			
	Yes	2.19	0.46	10.37	0.33	0.62	0.17	2.3	0.48
Employment	Employed	1				1			
	FT student	0.75	0.17	3.33	0.71	2.22	0.31	15.77	0.43
	Unemployed	0.94	0.25	3.51	0.93	0.43	0.09	2.06	0.29
	Retired, ill or housewife	8.81	2.06	37.64	**0.003**	0.27	0.06	1.21	0.09
**ETHNICITY & RELIGIOSITY**									
Ethnicity	Pakistani	1				1			
	Bangladeshi	1.24	0.46	3.29	0.67	0.32	0.11	0.94	**0.04**
Born in UK	No	1				1			
	Yes	0.22	0.08	0.65	**0.006**	0.52	0.14	1.91	0.33
Importance of religion in your life	Very	1				1			
	Fairly	0.67	0.18	2.48	0.54	0.08	0.01	0.48	**0.006**
	Not/don't know	0.36	0.04	2.89	0.33	0.07	0.01	0.81	**0.03**
Frequency of attending place of worship	Never	1				1			
	Monthly or less	1.42	0.37	5.52	0.41	0.65	0.16	2.6	0.54
	weekly or more	2.83	0.7	11.48	0.14	0.56	0.11	2.8	0.48
**PSYCHOSOCIAL ADVERSITY AND SOCIAL CAPITAL**									
Social capital score		0.63	0.5	0.8	**<0.001**	1	0.8	1.26	0.97
Total contacts in previous week		1.52	1.26	1.83	**<0.001**	0.95	0.73	1.24	0.7
Political Engagement Score		0.98	0.77	1.25	0.89	0.77	0.55	1.07	0.12
Threatening Life Events Score		1.13	0.67	1.9	0.64	0.47	0.2	1.12	0.09
Discrimination score		0.61	0.31	1.19	0.64	0.66	0.23	1.92	0.45
**HEALTH MEASURES**									
Anxiety score on GAD7		0.79	0.61	1.03	0.09	0.94	0.75	1.19	0.61
Depression score on PHQ9		1.22	1	1.48	**0.04**	1.33	1.03	1.72	**0.03**
Total Health Score		0.89	0.56	1.39	0.6	0.7	0.43	1.11	0.13

Compared with the group 2, people in the group showing most sympathies for violent protest and terrorism (group 3) were more likely to report depressive symptoms, less likely to include people of Bangladeshi ethnic origin, and less likely to include people reporting religion as fairly rather than very important in their lives. Low levels of political engagement were no longer associated with membership of group 3. Contrary to popular views about radicalisation, unemployment, educational achievements, discrimination, and stressful life events did not show associations with sympathies towards violent protest and terrorism.

Group 1 showed the most condemnation of violent protest and terrorism. Group 1 was more likely (compared with group 2) to include people unavailable for work (mostly women who were looking after the home) and migrants born outside of the UK who would therefore have migrated to the UK; Group 1 compared with 2, reported a greater number of social contacts, lower social capital, and marginally more depressive symptoms. In univariable analyses, fewer people in groups 1 were unemployed, compared with group 2. Theis findings were not sustained in multivariable analyses.

The PHQ score was entered as a continuous score of depressive symptoms. The PHQ score does not indicate clinically significant depressive symptoms and there may be threshold effects. In order to investigate the relationship between depression and cluster derived group membership further, we applied validated clinical thresholds used to screen for mild (5 or more) and moderate (10 or more) depression. On repeating the analyses, mild depression was associated with membership of group 3 but moderate depression was not (see Table 3). If depression was entered as a binary variable using a threshold of 5 or more to indicate mild and moderate depressive symptoms, depression was still more prevalent in group 3. A log likelihood ratio test indicated no difference in model fit between a 3 (none, mild, moderate) or 2 category (none, mild and more severe) measure of depression (X2 = 2.91, df = 2, p = 0.23). Although gender was not associated with cluster membership, the point estimates indicate women were less likely to be in group 1 and more likely to be in group 3; and given depression is more common in women, this model was repeated with an interaction term between gender and depression. This model did not show a better fit (likelihood ratio test X2 = 4.44, df = 2, p = 0.11); the findings overall remained identical with a non-significant interaction term. In order to assess whether the suicide item in the PHQ9 was driving the associations with depression, we repeated these models without this item and found the same overall findings.

**Table 3:** Association of Depression category with cluster membership (fully adjusted model).

		Cluster 1			Cluster 3	
Depression Category	RR	95%CI	P value	RR	95%CI	p value
None (PHQ score 0–4)	1			1		
Mild (PHQ score 5–9)	1.53	0.34 to 6.87	0.58	5.22	1.33 to 20.54	**0.02**
Moderate (PHQ score > or = 10)	9.54	0.43 to 210.34	0.15	6.52	0.35 to 121.78	0.21
None (PHQ score 0–4)	1			1		
Mild or moderate (PHQ score 5 or more)	2.03	0.47 to 8.76	0.34	5.43	1.35 to 21.84	**0.02**

Cluster 2 is reference.

Repeating the analysis after removing the suicide item from the PHQ9 measure of depression produces the same overall findings.

## Discussion

### Principal findings

Depressive symptoms (PHQ score of 5 or more indicating at least mild depression) were more common among those showing the most sympathy towards violent protest and terrorism. Although previous studies of terrorists have not shown significantly greater levels of severe mental illness (for example, psychoses with delusions and hallucinations), small studies of convicted terrorists and of teenagers in Palestine have suggested depressive and anxiety symptoms are important [23] [13]. One study seeking to define religious terrorism proposed the presence of ‘dogma induced psychotic depression’ but this investigation was not based on a structured diagnostic or screening instrument. The place of psychological distress in terrorism is controversial [48]. It is known that low mood is associated with hopelessness about the future, and suicidal feelings, perhaps mediated through feelings of low self-esteem, cognitive distortions in weighing up everyday events and experiences as more negative [38]. These cognitive biases may be seen as adaptive if they reflect social and economic injustices, and experiences of structural violence; or these may reflect depressive illness if there is not a history of such adversity. Our findings suggest the latter, that depressive symptoms independent of psychosocial adversity were associated with sympathies towards violent protest and terrorism.

More generally, depression has been implicated in acts of aggression [49], [50]. Although depression and aggression can be present from an early age and have genetic and neurochemical origins [50], [51], depression can also be a consequence of chronic adversity, and can lead to maladaptive behaviour, social strain [33] or abnormal personality development [51]. Personality disorder is often implicated in crime, but is an unlikely explanation of our finding as the majority of our sample were in employment and reported active social networks (and additionally did not report frequent hospital attendance due to accidents and injuries; unreported data).

Social isolation has been proposed as a risk factor for radicalization [52], and is also implicated as a link between biological mechanisms of aggression and depression when tested in animal models [53]. Consistent with this perspective, we found that the group showing the strongest condemnation appeared to have more social contacts. Isolation from the wider community, together with feelings of identification and cohesion within terrorist organisations, are a proposed mechanism by which people are persuaded to take up violence, under the influence of newly formed radical affiliations [12], [19]. In multivariate analyses, women seemed more likely to be in the most sympathetic group, indicating greatest risk; this finding did not reach statistical significance. A higher risk for women has previously been reported but only where social isolation, powerlessness and oppression limited alternative opportunities or lifestyles [54]. More in-depth qualitative work exploring Muslim women's experiences of disadvantage and oppression may offer useful insights into the place of gender politics and gender disadvantage as mechanism of radicalisation.

A surprising finding was that low levels of social capital were not related to sympathies towards terrorism but were associated with greater condemnation of terrorist acts. In our study, social capital was measured by satisfaction with residential area, trust in neighbours and feelings of safety. As expected in accord with the wider literature, poor social capital was associated with depression (unreported data), suggesting that the survey questions seem to tap the appropriate concepts [55]. A low score, therefore, reflected fears associated with the neighbourhood, including violence in the community, and explains condemnation of perceived threats. This finding is also consistent with a recent analysis suggesting that higher social capital can actually foster the formation of terrorist groups through greater opportunities for co-operation and by the exploitation of altruistic aspirations in a open democratic political system [27]. However, a study of social capital and terrorism, conducted over 12 years in 150 countries, showed that as well as fostering more terrorist organisations, higher social capital actually correlated with fewer terrorist attacks. So the influence of social capital is complex and it does not seem to be easily modified for predictable preventive effects.

### Strengths and Weaknesses

Although the three-group solution was selected by a statistically acceptable method of cluster analysis using a variety of sensible criteria to indicate the ‘correct’ number of groups, the clusters derived should be regarded as a preliminary and useful basis for understanding and describing the survey responses. Replication is recommended as one study does not provide definitive conclusions on which to base policy.

As a cross-sectional study, causality cannot be directly inferred but the three groups derived from cluster analysis are akin to an ordinal variable permitting the comparison of specific characteristics across the three groups. However, no single variable showed linear trends across all three clusters. This suggests protective factors associated with condemnation are different to those associated with the development of sympathies for violent protest and terrorism. Alternatively, we may have not included other factors that might be linearly related with developing sympathies for radicalisation.

Importantly, this study does not show which people who are sympathisers are likely to progress to terrorist acts. However, there is a sequence of events consistent with the stages of radicalization [35], each with specific contexts and risk of progression [7]. This model of public health epidemiology recommends that those ‘infected’ with radical ideology, or already radicalised, need to be isolated so that they cannot influence those around them. The criminal justice system fulfils this function. Those who are vulnerable require inoculation through a wider set of ideas, including orthodox religious texts that decry homicide and terrorism. Furthermore, social networks promote resistance by offering a range of cultural identities and opportunities and this in itself may be protective; for example, integrated cultural identities protect against psychological distress in young people by offering bridging social capital across contrasting identity groups [56]. Studies of resilience to such radicalising ideas, where resilience consists of resistance despite exposure to radicalising ideas in the presence of SVPT, are needed.

Another reason for studying and preventing SVPT in the population is that these become part of the political rhetoric of terrorists and are used to justify their actions. Through encouraging SVPT, terrorists and extremists can seek resources for their cause. There may be communalities with other extremist movements, for example, animal rights protesters, or in situations of war and conflict, or where extreme right wing parties target particular ethnic, racial or religious groups [57], [58]. Indeed, the boundaries between protest movements and terrorism require further investigation.

We had interpreted condemnation of sending British troops to conflict zones as consistent with radicalisation because terrorists have reported such actions as justification for their acts [4]. We generated our items to measure radicalisation by reference to such literature and by focus groups assuring us of face and content validity. However, such condemnation could also indicate a position of conscientious objection. In people of Muslim heritage, this stance might be misunderstood to indicate radicalisation rather than an anti-war or pacifist political position. Future studies will need to carefully discern whether such political objections are separate from beliefs that are on the pathway to radicalisation. We did not find political engagement to be associated with radicalisation suggesting that our findings are unlikely to reflect political activism or conscientious objection.

Terrorist organisations in the past and in other cultures were motivated by different social and political factors. As a result, our findings relate to the current priorities of counter-terrorism in Europe and North America, namely of understanding and preventing the radicalisation of home-grown youth. It would not be prudent to generalise the findings to other contexts and types of terrorism, although similar research is feasible in other contexts. For example, although ‘psychotic depression’ was implicated in a study undertaken in a conflict zone [24], our findings indicate a modest effect of depressive symptoms; we did not measure clinical symptoms of psychosis directly. The attribution of psychosis defined on the basis of delusions is subject to criticism, as delusions are culturally shaped and can be seen as psychopathological only if the beliefs held are culturally inappropriate. Fear of the enemy, paranoia and depressive symptoms may be seen as ordinary responses to war and conflict. Thus the study of psychological distress in future studies must consider context: the influence of conflict, culture and war on SVPT. Furthermore, our finding suggest that depressive symptoms may be both protective and risk factors, but it was only depressive symptoms meeting a screening threshold for mild depressive illness that were risk factors. The place of depressive symptoms clearly warrants further investigations.

### Implications

Terrorist acts not only result in death, illness, and severe injury to members of the public and emergency services, they also impact adversely on social cohesion, accentuating divisions between different racial and religious groups [59]–[61]. They raise legitimate fears about safety and security [61], [62]. Trauma, multiple bereavements, and fear can have long-term consequences for psychological health [63]. A preventive approach to radicalisation is not part of current UK counter-terrorism policy, which focuses on those likely to commit terrorist acts. Our study shows that there are modifiable risk and protective factors for the earliest stage on the pathway to violent protest. The potential benefits of disrupting the pathways to radicalisation go beyond security issues and have implications for preventing significant depressive symptoms, promoting wellbeing and perhaps social capital [64]. More research is needed into the causes of depressive thinking implicated in our study. Future studies must investigate the place of different types of social assets, depressive symptoms, and psychosocial adversity in the process of radicalisation in different heritage groups and in different country and regional contexts. Our methods do offer an alternative paradigm for testing interventions aimed at preventing or reversing the early stages of violent radicalisation.

### Key Messages

- Studies of sympathies for terrorism and violent radicalisation are needed and feasible to undertake in a Muslim minority country.
- Mild depressive symptoms as assessed on the PHQ9 are associated with sympathies for violent protest and terrorism.
- A greater number of social contacts and being a migrant were associated with more condemnation. Poorer social capital and being unavailable for work because of housewife roles and disability were associated with condemnation.
- Future work needs to investigate whether standardised measures of social capital to replicate this unexpected finding and to help understand the mechanisms.

## Supporting Information

Table S1 **Proportion (as %) of respondents endorsing each Likert response of the 16 Radicalization items.**
(DOCX)

Table S2 **Means scores for each item and all items by clusters.**
(DOCX)
