# Supplementary material for: Might Depression, Psychosocial Adversity, and Limited Social Assets Explain Vulnerability to and Resistance against Violent Radicalisation?
Source: PLoS One. 2014 Sep 24;9(9):e105918. doi: 10.1371/journal.pone.0105918 (PMC4174521; doi:10.1371/journal.pone.0105918)
Supplement: Table S1 — Proportion (as %) of respondents endorsing each Likert response of the 16 Radicalization items. (DOCX) [file pone.0105918.s001.docx]

| **Table S1: Proportion (as %) of respondents endorsing each Likert response of the 16 Radicalization items** | | | | | | | | | | | | | | | | |
| --- | --- | --- | --- | --- | --- | --- | --- | --- | --- | --- | --- | --- | --- | --- | --- | --- |
|  |  | **Un-weighted data** | | | | | | |  | **Weighted data** | | | | | | |
|  |  |  |  |  |  |  |  |  |  |  |  |  |  |  |  |  |
|  |  | **Condemn** | | | ***Undecided*** | **Sympathise** | | |  | **Condemn** | | | ***Undecided*** | **Sympathise** | | |
| N=608 for all items |  | ***Completely*** | ***To some extent*** | ***A little*** |  | ***A little*** | ***To some extent*** | ***Completely*** |  | ***Completely*** | ***To some extent*** | ***A little*** |  | ***A little*** | ***To some extent*** | ***Completely*** |
|  |  | % | % | % | % | % | % | % |  | % | % | % | % | % | % | % |
| **People who:** |  |  |  |  |  |  |  |  |  |  |  |  |  |  |  |  |
| **Take part in non-violent political protest** |  | 14.7 | 9.3 | 5.7 | 21 | 8.8 | 16.2 | 24.3 |  | 14.31 | 9.04 | 5.6 | 23 | 8.65 | 15.79 | 23.69 |
| **Minor crime in political protests** |  | 62.5 | 15.4 | 7.5 | 11.1 | 0.8 | 2 | 0.7 |  | 61.35 | 15.13 | 7.4 | 12.67 | 0.82 | 1.97 | 0.66 |
| **Use violence in political protest** |  | 73.6 | 9.5 | 3.9 | 10 | 1.2 | 1.2 | 0.7 |  | 72.37 | 9.38 | 3.78 | 11.51 | 1.15 | 1.15 | 0.66 |
| **Threats of terrorist actions as part of political protest** |  | 79.7 | 5.2 | 2.8 | 10.3 | 1.3 | 0.2 | 0.5 |  | 78.62 | 5.1 | 2.8 | 11.51 | 1.31 | 0.17 | 4.93 |
| **Organize radical groups but do not participate** |  | 79.2 | 6.2 | 4.2 | 8.9 | 0.5 | 0.7 | 0.3 |  | 77.8 | 6.09 | 4.11 | 10.52 | 0.49 | 0.66 | 0.33 |
| **Commit terrorist actions as form of political protest** |  | 79.8 | 4.9 | 2.5 | 11.7 | 0.5 | 0.2 | 0.5 |  | 78.45 | 4.77 | 2.47 | 13.16 | 4.93 | 0.17 | 0.49 |
|  |  |  |  |  |  |  |  |  |  |  |  |  |  |  |  |  |
| **The following actions:** |  |  |  |  |  |  |  |  |  |  |  |  |  |  |  |  |
| **Violence to protect your family** |  | 33.2 | 11.2 | 5 | 20.4 | 4.2 | 13.1 | 12.9 |  | 32.57 | 11.02 | 4.93 | 21.88 | 4.11 | 12.83 | 12.67 |
| **Violence organized by groups to protect your own race, religion, tribe** |  | 54.1 | 11 | 4.2 | 20.6 | 4.2 | 3 | 2.9 |  | 52.63 | 10.69 | 4.11 | 22.7 | 4.11 | 2.96 | 2.8 |
| **Violence to fight injustice by the police** |  | 58.5 | 10.3 | 6.1 | 17.9 | 3.5 | 1.9 | 2 |  | 57.24 | 10.03 | 5.92 | 19.57 | 3.45 | 1.81 | 1.97 |
| **Violence to fight injustice by governments** |  | 60.4 | 9.1 | 6 | 17.3 | 3.7 | 1.9 | 1.7 |  | 59.21 | 8.88 | 5.92 | 18.92 | 3.62 | 1.81 | 1.65 |
| **Bombs to fight injustice** |  | 78.8 | 4.9 | 3.4 | 11.8 | 0.7 | 0.2 | 0.3 |  | 76.81 | 4.77 | 3.29 | 13.98 | 0.66 | 0.17 | 0.33 |
| **Suicide bombs to fight injustice** |  | 80.3 | 4.6 | 2.4 | 12 | 0.2 | 0.3 | 0 |  | 78.45 | 4.44 | 2.3 | 14.31 | 0.17 | 0.33 | 0 |
| **British Government sending troops to Afghanistan*** |  | 47.86 | 14.47 | 6.58 | 23.68 | 1.64 | 3.13 | 2.63 |  | 49.53 | 13.15 | 12.11 | 23.21 | 0.58 | 0.72 | 0.71 |
| **British Government sending troops to Iraq*** |  | 49.18 | 12.99 | 6.25 | 25.33 | 1.48 | 2.3 | 2.47 |  | 49.48 | 11.52 | 9.96 | 26.77 | 0.95 | 0.67 | 0.65 |
| **People in Britain going to fight in Afghanistan** |  | 51.97 | 10.86 | 4.11 | 25.83 | 3.13 | 2.3 | 1.81 |  | 45.06 | 12.48 | 7.84 | 28.51 | 3.41 | 1.65 | 1.05 |
| **People in Britain going to fight in Iraq** |  | 50.99 | 9.38 | 4.44 | 27.96 | 2.63 | 2.14 | 2.47 |  | 44.07 | 10.32 | 6.97 | 33.09 | 1.77 | 1.79 | 2 |
|  |  |  |  |  |  |  |  |  |  |  |  |  |  |  |  |  |
| * Reverse scored in summing total score as sympathy here supports British government support and not a radical stance | | | | |  |  |  |  |  |  |  |  |  |  |  |  |
