# Supplementary material for: Might Depression, Psychosocial Adversity, and Limited Social Assets Explain Vulnerability to and Resistance against Violent Radicalisation?
Source: PLoS One. 2014 Sep 24;9(9):e105918. doi: 10.1371/journal.pone.0105918 (PMC4174521; doi:10.1371/journal.pone.0105918)
Supplement: Table S2 — Means scores for each item and all items by clusters. (DOCX) [file pone.0105918.s002.docx]

| **Table S2: Means scores for each item and all items by clusters** | | | | |
| --- | --- | --- | --- | --- |
|  |  |  |  |  |
| **16 Radlicalisation Items** | **Cluster 1** | **Cluster 2** | **Cluster3** |  |
|  | **(N=93)** | **(N=423)** | **(N=92)** |  |
| **People who:** |  |  |  |  |
|  |  |  |  |  |
| ***Take part in non-violent political protest*** | -0.2 | 0.49 | -0.61 |  |
|  |  |  |  |  |
| ***Minor crime in political protests*** | -3 | -2.27 | -0.65 |  |
|  |  |  |  |  |
| ***Use violence in political protest*** | -3 | -2.45 | -0.37 |  |
|  |  |  |  |  |
| ***Threats of terrorist actions as part of political protest*** | -3 | -2.8 | -0.37 |  |
| ***Organize radical groups but do not participate*** | -3 | -2.63 | -0.38 |  |
|  |  |  |  |  |
| ***Commit terrorist actions as form of political protest*** | -2.88 | -2.72 | -0.11 |  |
|  |  |  |  |  |
| **The following actions:** |  |  |  |  |
| ***Violence to protect your family*** | -3 | -0.38 | -0.18 |  |
|  |  |  |  |  |
| ***Violence organized by groups to protect your own race, religion, tribe*** | -3 | -1.61 | -0.21 |  |
|  |  |  |  |  |
| ***Violence to fight injustice by the police*** | -3 | -1.57 | -0.27 |  |
|  |  |  |  |  |
| ***Violence to fight injustice by governments*** | -3 | -1.82 | -0.04 |  |
|  |  |  |  |  |
| ***Bombs to fight injustice*** | -3 | -2.59 | -0.36 |  |
|  |  |  |  |  |
| ***Suicide bombs to fight injustice*** | -3 | -2.7 | -0.36 |  |
|  |  |  |  |  |
| ***British Government sending troops to Afghanistan**** | 3 | 2.03 | 0.28 |  |
| ***British Government sending troops to Iraq**** | 3 | 1.99 | 0.15 |  |
|  |  |  |  |  |
| ***People in Britain going to fight in Afghanistan*** | -3 | -1.68 | -0.19 |  |
| ***People in Britain going to fight in Iraq*** | -2.99 | -1.61 | -0.05 |  |
|  |  |  |  |  |
|  | -33.63 | -22.32 | -3.64 |  |
| **Sum of mean scores for all items** |  |  |  |  |
|  |  |  |  |  |
| * reverse scored |  |  |  |  |
